# Quality of recovery (QoR-15) after patient-controlled sedation during venous port implantation: A prespecified substudy of the PACSPI-2 randomised clinical trial

**DOI:** 10.1371/journal.pone.0360479

**Published:** 2026-10-14

**Authors:** Stefanie Seifert, Andreas Nilsson, Josip Azman, Olle Eriksson, Michelle S. Chew, Fredrik Hammarskjöld, Knut Taxbro

**Affiliations:** 1 Department of Anaesthesia and Intensive Care Medicine, Ryhov County Hospital, Jönköping, Sweden; 2 Department of Biomedical and Clinical Sciences, Linköping University, Linköping, Sweden; 3 Department of Anaesthesia and Intensive Care, Linköping University Hospital, Linköping, Sweden; 4 Department of Medical and Health Sciences, Linköping University, Linköping, Sweden; 5 Pain and Rehabilitation Department, Linköping University Hospital, Linköping, Sweden; 6 Futurum, Academy of Health and Care, Jönköping, Sweden; 7 Department of Perioperative Medicine and Intensive Care, Karolinska University Hospital, Stockholm, Sweden; Universidade de Trás-os-Montes e Alto Douro: Universidade de Tras-os-Montes e Alto Douro, PORTUGAL

## Abstract

**Background:**

Postoperative recovery after subcutaneous venous port implantation is generally considered uncomplicated; however, patient-reported recovery outcomes in patients with cancer remain poorly characterized. The impact of patient-controlled sedation (PCS) on postoperative recovery has not been established.

**Objective:**

To assess early postoperative recovery using the Quality of Recovery-15 (QoR-15) questionnaire in a prespecified substudy of the PACSPI-2 randomised clinical trial and to compare recovery between patients receiving local anaesthesia alone and those receiving local anaesthesia combined with patient-controlled sedation.

**Design and setting:**

Prespecified substudy of a randomised, open-label clinical trial conducted at two Swedish anaesthesia departments.

**Patients:**

Three hundred thirty-four adult patients with cancer.

**Interventions:**

Local anaesthesia (LA) alone or LA combined with propofol-alfentanil PCS during SVP implantation.

**Main outcome measures:**

Primary outcome was difference of postoperative total QoR-15 scores between groups. The primary endpoint of the parent PACSPI-2 trial was maximal intraprocedural pain.

**Results:**

There was no overall difference in total postoperative QoR-15 scores between the treatment groups (P = 0.61). Total QoR-15 scores changed significantly over time (P < 0.001) in both groups. By postoperative day 1 (POD 1), mean QoR-15 scores had increased from baseline in both groups (mean difference 2.9 and 3.1, respectively; P = 0.02 for both), although these changes were below the published minimal clinically important difference (MCID) of 6. Before discharge, the LA + PCS group showed its largest within-group improvement in QoR-15 (mean difference 5.6, P < 0.001), primarily driven by the “Emotional state” and “Psychological support” dimensions, however no significant between group differences were observed. Median pain scores on POD 1 were 3 (IQR 2–5) in both groups, with NRS ≥ 4 reported by 49% in the LA and 48% in the LA + PCS group.

**Conclusions:**

Early postoperative recovery after subcutaneous venous port implantation was generally good and did not differ between the LA and LA + PCS group. A transient within-group improvement in emotional and psychological recovery domains was observed before discharge in the LA + PCS group. Pain on POD 1 remained common in both groups. These findings were based on secondary analyses and should therefore be considered exploratory.

**Trial registration:**

European Union Clinical Trials Registry: EudraCT: 2021-003821-31, ClinicalTrials.gov: NCT05688384

## Introduction

Cancer remains one of the most prevalent global diseases, with a substantial proportion of patients requiring long-term venous access for systemic therapy [1]. Subcutaneous venous ports (SVP) are the recommended central venous access devices for a majority of cancer patients [2,3]. They are widely used to facilitate intravenous chemotherapy, blood sampling, and medication administration and represent one of the most commonly performed minor surgical procedures in oncology care in Sweden [4].

Although minimally invasive, SVP implantation can cause pain and anxiety in vulnerable patients. No procedure-specific recommendations regarding pain management exist and clinical practices vary widely. The procedure is typically performed in an ambulatory setting, with patients discharged within minutes to hours after the procedure.

Traditional postoperative outcomes focus on morbidity, mortality, and pain management, however these measures capture only part of the recovery. Quality of Recovery-15 (QoR-15), a validated patient-reported outcome measure (PROM) [5], reflects the patients´ ability to resume normal activities and is an important indicator of a successful perioperative experience [6]. Fifteen questions assess five domains of patient-reported health status: pain, physical comfort, physical independence, psychological support and emotional state. An 11-point numerical rating scale leads to a minimum score of 0 (very poor recovery) and a maximum score of 150 (excellent recovery). The minimal clinically important difference (MCID) describes the smallest change in score that constitutes a meaningful change in health status and is recommended to be a value of 6 [7,8]. Growing evidence supports the use of PROMs to improve patient-centred care and uncover underreported issues in cancer settings [9]. Recent research further recommends incorporating quality of recovery as an endpoint in perioperative clinical trials [10,11]. The QoR-15 instrument has been evaluated in day case surgery [6]. The Swedish version of QoR-15 has been validated [12].

Despite the perception that postoperative recovery after SVP implantation is good, there are no data on patient-related outcomes in the early postoperative period. Additionally, the impact of intraoperative analgesia and sedative management on postoperative recovery has not been studied. Although SVP implantation is minimally invasive, patient-reported recovery data after discharge are sparse and may reveal unrecognized pain or distress.

This study, a prespecified substudy of the PACSPI 2 trial [13], assessed postoperative recovery in cancer patients receiving either local anaesthesia alone (LA) or in combination with patient-controlled sedation (PCS) with propofol and alfentanil (LA + PCS) for SVP implantation. Patient recovery was measured using the QoR-15 questionnaire at three distinct time points: before surgery (baseline), postoperatively prior to discharge (postop), and on the first postoperative day (POD 1) via telephone interview. We hypothesised that postoperative QoR-15 scores would be similar between patients receiving LA alone and those receiving LA + PCS.

## Methods

### Trial design, approval, registration and oversight

This study was a prespecified substudy of the PACSPI-2 randomised, open-label clinical trial, conducted at two Swedish anaesthesia departments (Linköping University Hospital and Ryhov County Hospital, Jönköping). It reports a prespecified secondary analysis of postoperative recovery assessed using the QoR-15 questionnaire [13]. Ethical approval was provided by the Swedish Ethical Review Authority (Dnr 2022-04888-01/ 02), on February 8, 2022. The Swedish Medical Products Agency (Dnr: 5.1-2022-2057) gave its approval on 15 march 2022. The parent trial PACSPI-2 was conducted according to the Declaration of Helsinki, adhered to Good Clinical Practice and the CONSORT guidelines and was registered at clinicaltrials.gov (NCT 05688384). All participants provided written informed consent. Independent site monitoring visits were undertaken throughout the trial period, and formal data validation procedures were completed before statistical analyses commenced. The study protocol and statistical analysis plan are provided as Supporting Information (S1 and S2 File).

### Patient population, inclusion, and exclusion criteria

Adult patients aged ≥18 years with a confirmed cancer diagnosis scheduled for SVP implantation were considered eligible for inclusion. Exclusion criteria included: inability to operate the PCS device, insufficient proficiency in Scandinavian languages, requirement for general anaesthesia, contraindications to sedation assessed by the attending anaesthesiologist, non-fasting status, absence of suitable peripheral intravenous access (PVC), pregnancy, or previous enrolment in the study.

### Randomisation

Participants were allocated to receive either LA or LA with PCS in a 1:1 ratio. Randomisation was performed using a secure, web-based electronic data capture system (REDCap, Linköping University) [14]. The allocation sequence was produced by an independent statistician using variable block sizes. Allocation concealment was maintained, however due to the nature of the intervention, neither clinical personnel nor participants were blinded to the group assignment.

### Interventions

Eligible patients were randomised to either a control group of LA for SVP-implantation or an intervention group of PCS with propofol and alfentanil as adjuncts to LA for SVP-implantation.

All patients received infiltration of LA at the surgical site, and the type and dosage determined by the attending anaesthesiologist.

In the intervention group, patients received instruction on operating the PCS pump (Syramed mSP6000; Arcomed AG, Kloten, Switzerland). The pump contained 36 ml of propofol (10 mg ml^-1^) and 4 ml of alfentanil (0.5 mg ml^-1^). Each patient-initiated activation delivered a 0.5 ml bolus – equivalent to 4.5 mg of propofol and 0.025 mg of alfentanil – administered over 10 seconds. The system allowed up to six boluses per minute, with a maximum delivery of 27 mg propofol and 0.15 mg alfentanil per minute, without lockout interval. Patients were permitted to begin using PCS immediately after establishing a PVC and before skin disinfection.

### Timing of assessments

The QoR-15 questionnaire was administered at three predefined time points: preoperatively (baseline) in the waiting area after inclusion into the study, postoperatively in the recovery unit prior to discharge, and 24 hours later on postoperative day 1 (POD 1) through telephone interview. POD 1 was selected as the follow-up time point because the optimal timing for detecting postoperative pain–related recovery issues is uncertain. This early assessment was intended to capture potential pain problems before they might be overlooked at later time points. Study nurses administered the questionnaire by reading the questions aloud and documenting the patients´ responses to the CRF.

Standard monitoring included non-invasive blood pressure measurements, heart rate, oxygen saturation and respiratory rate throughout the procedure.

All SVP implantations were performed by anaesthesiologists using the percutaneous Seldinger technique. Intra-procedural sedation depth was evaluated using the Observer’s Assessment of Alertness/Sedation (OAA/S) scale, a validated six-level sedation scoring instrument ranging from 5 (fully alert to verbal stimulus) to, 4 (slowed response to name), 3 (response only to loud or repeated verbal stimulus), 2 (response to gentle physical stimulation), 1 (response to painful stimulation), and 0 (no response to painful stimulation) [15]. Sedation levels were recorded at four predefined procedural time points: preoperative skin antisepsis (T1), during local anaesthetic injection (T2), during catheter tunnelling (T3), and at removal of sterile drapes (T4).

### Endpoints

Primary endpoint

Primary outcome was difference of postoperative total QoR-15 scores between groups.

Secondary endpoints

Several secondary endpoints were evaluated: postoperative QoR-15 domain scores, change over time, postoperative QoR-15 item scores, postoperative pain scores and postoperative consumption of analgesics.

### Sample size and statistical analysis

The sample size of the parent PACSPI-2 trial was determined by the primary endpoint of maximal intraprocedural pain. For the present prespecified secondary analysis, the available sample size was evaluated against the established MCID of 6 points for the QoR-15 score [8]. The calculation was based on a two-sided comparison of two independent means assuming a standard deviation of 19, 80% power, and a two-sided significance level of 0.05. Under these assumptions, a total sample size of 314 participants was required to detect the predefined MCID.

Continuous variables are presented as mean (standard deviation [SD]) or median (interquartile range [IQR]), as appropriate. Categorical variables are presented as counts and percentages. Between-group comparisons of continuous variables were performed using the Mann–Whitney U test. Categorical variables were analysed using Pearson’s chi-square test or Fisher’s exact test, as appropriate.

Repeated QoR-15 measurements were analysed using the General Linear Model–Univariate procedure in IBM SPSS version 29 (IBM Corp., Armonk, NY, USA). Total QoR-15 score was specified as the dependent variable, with treatment group and assessment time included as fixed factors and patient ID as random factor. The model included the main effects of treatment group and time, treatment group-by-time interaction and patient ID within treatment group. Estimated marginal means with 95% confidence intervals (CIs) were obtained, and pairwise comparisons between time points were performed using the least significant difference (LSD) procedure.

Primary and secondary analyses were based on the intention-to-treat population. Repeated QoR-15 analyses included all randomised participants with available outcome data. Participants contributed all available QoR-15 measurements; observations with missing QoR-15 values were excluded from the repeated-measures analyses. Consequently, these analyses represent available-case analyses within the intention-to-treat population. A complete-case sensitivity analysis including only participants with QoR-15 data available at all three assessment time points was performed. To assess the potential impact of missing outcome data, a supplementary analysis compared baseline characteristics between participants with complete and incomplete follow-up. (S3 File)

No adjustment for multiple comparisons was performed. Analyses of QoR-15 domains and individual items should therefore be regarded as exploratory. A two-sided P value <0.05 was considered statistically significant.

## Results

Between January 19, 2023 and November 8, 2024, 791 patients were evaluated for study inclusion. Among these, 109 did not meet the eligibility criteria, 349 chose not to participate, and 340 were subsequently randomised. Six patients were identified as having been randomised despite fulfilling exclusion criteria, and excluded. The final study population comprised 334 patients, of whom 168 received local anaesthesia (LA) alone and 166 received LA combined with patient-controlled sedation (LA + PCS) (Fig 1). SVP implantation was unsuccessful in one patient in the LA group, resulting in missing primary outcome data. Follow-up on POD 1 was incomplete in 16 (9.6%) patients in each group. None of the participants withdrew their consent during the study period.

**Fig 1:**
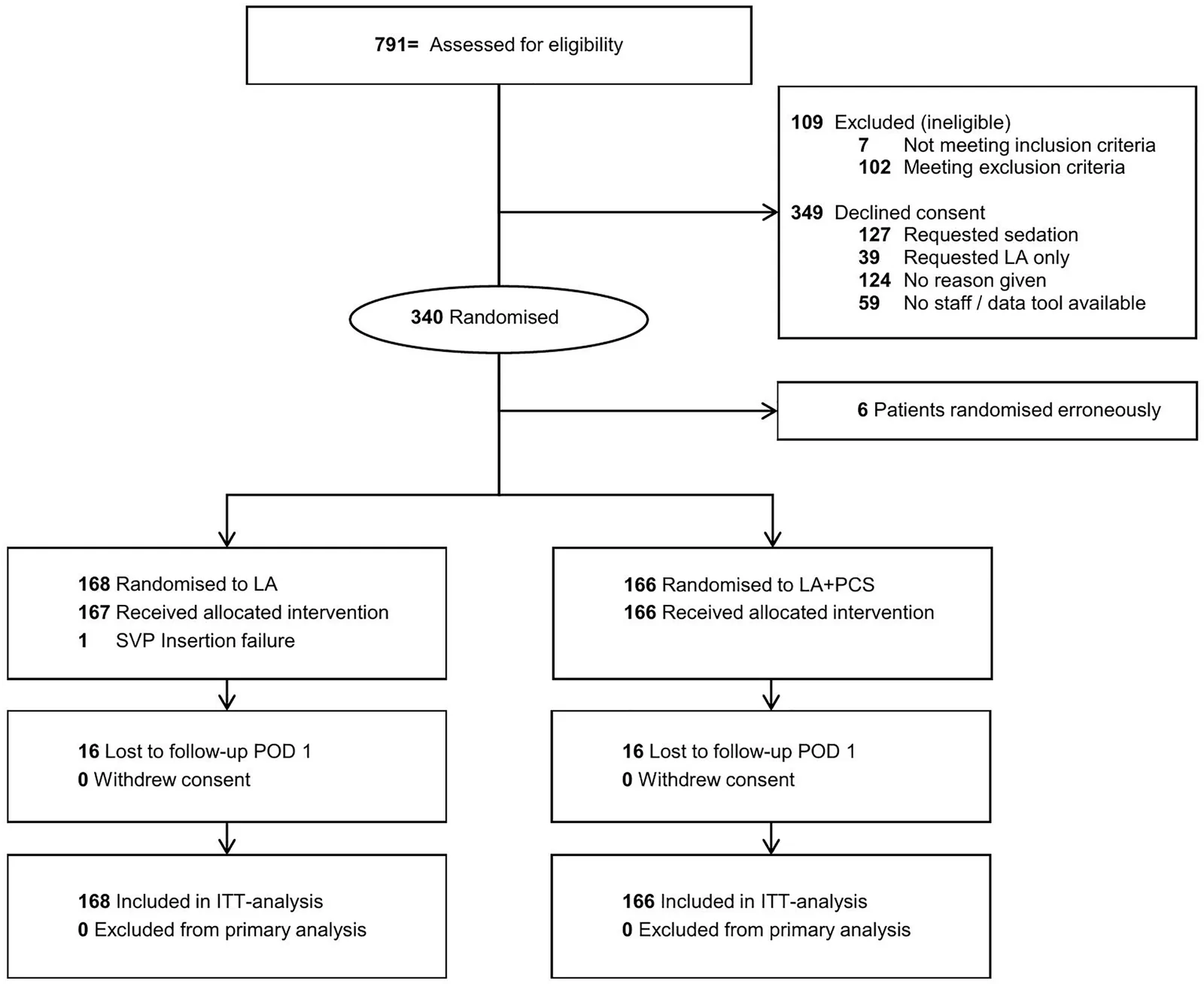
CONSORT flow diagram of participant recruitment, randomization, follow-up, and analysis.

The clinical and procedural characteristics are summarised in Tables 1 and 2, respectively. The median age of the patients was 70 (61−76) years, and 173 (51.8%) were male. Most SVP´s were inserted under ultrasound guidance (332, 99.7%) into the right (300, 89.8%) jugular vein (327, 98.2%). Patients in the LA + PCS group achieved deeper levels of sedation at procedural time points T2 (OR 0.32; 95% CI 0.14–0.71; P = 0.003) and T3 (OR 0.21; 95% CI 0.10 to 0.41; P < 0.001) than patients in the LA group.

**Table 1:** Clinical characteristics of patients.

	LA (n = 168)	LA + PCS (n = 166)
Age (year), median (IQR), [No]	68.0 (60-76) [168]	72.0 (60-77) [166]
Sex, No. (%) Female Male	168 80 (47.6) 88 (52.4)	166 81 (48.8) 85 (51.2)
ASA physical status, No. (%) 1 2 3 4	166 7 (4.2) 94 (56.6) 64 (38.6) 1 (0.6)	165 9 (5.5) 77 (46.7) 79 (47.9) –
BMI, median (IQR), [No]	24.6 (22.7-28.2), [168]	25.1 (22.1-28.3), [165]
Cancer type No. (%) Haematological Breast Colorectal Upper GI-tract Urogenital Lung ENT Other	167 14 (8.4) 21 (12.6) 35 (21.0) 33 (19.8) 32 (19.2) 13 (7.8) 5 (3.0) 14 (8.4)	165 17 (10.3) 13 (7.9) 36 (21.8) 35 (21.2) 33 (20.0) 12 (7.3) 2 (1.2) 17 (10.3)
Treatment strategy No. (%) Adjuvant Palliative	166 82 (49.4) 84 (50.6)	165 81 (49.1) 84 (50.9)
Premedication No. (%) No premedication Premedication Paracetamol NSAID Other	168 75 (44.6) 93 (55.4) 85 (91.4) 2 (2.2) 6 (6.5)	166 79 (47.6) 87 (52.4) 77 (88.5) 3 (3.4) 7 (8.0)
Centre No. (%) Jönköping Hospital Linköping Hospital	168 86 (51.2) 82 (48.8)	166 76 (45.8) 90 (54.2)

Abbreviations: IQR, interquartile range; LA, local anaesthetic; PCS, patient-controlled sedation; y, years; No, Number; n, sample size; ASA, American Society of Anaesthesiology; GI, gastrointestinal; ENT, ear-nose-throat; NSAID, nonsteroidal anti-inflammatory drug

**Table 2:** Insertion and sedation characteristics.

	LA (n = 168)	LA + PCS (n = 166)	P value
Insertion vein, No. (%) Jugular vein Subclavian vein Axillary vein	167 165 (98.8) 2 (1.2) 0 (0%)	166 162 (97.6) 3 (1.8) 1 (0.6)	
Laterality, No. (%) Right Left	168 144 (85.7) 24 (14.3)	166 156 (94.0) 10 (6.0)	
Ultrasound use, No. (%) No ultrasound used Ultrasound used	168 – 168 (100)	165 1 (0.6) 164 (99.4)	
Catheter type, No. (%) 8.5 Fr, small chamber 8.5 Fr, standard chamber Other	167 85 (50.9) 74 (44.3) 8 (4.8)	166 88 (53.0) 69 (41.6) 9 (5.4)	
Local anaesthetic type, No. (%) Mepivacaine 1% Mepivacaine 0.5% Other	168 87 (51.8) 79 (47.0) 2 (1.2)	166 83 (50.0) 83 (50.0) –	
Local anaesthetic volume (ml), median (IQR),[No.]	30 (20-38) [167]	27 (20-35) [164]	
Operating conditions, No. (%) 1 perfect conditions 2 minor impact on conditions 3 major impact on conditions 4 procedure cannot be performed	167 137 (82.0) 25 (15.0) 4 (2.4) 1 (0.6)	166 160 (96.4) 6 (3.6) – –	< 0.001
Sedation volume (ml), median(IQR) [No.] Alfentanil delivered mg Propofol delivered mg	0 (0−0) [165] 0 (0−0) 0 (0−0)	6 (3.5-9.5) [166] 0.3 (0.17-0.47) 54 (31.5-85.5)	< 0.001
Rescue sedation given No. (%) Not given Given Patient requested Operator requested Other	168 153 (91.1) 15 (8.9) 10 (66.7) 3 (20) 2 (13.3)	165 164 (99.4) 1 (0.6) – – 1 (100)	< 0.001
Rescue sedation type, No. (%) Propofol/Alfentanil Propofol Alfentanil Other	15 9 (60) 4 (26.7) 2 (13.3) –	1 – – – 1 (100)	
OAA/S T1, preop skin antisepsis, No (%) 5 awake 4 lethargic to normal tone 3 lethargic to loud tone 2 response mild prodding 1 response painful stimulus 0 no response painful stimulus	168 167 (99.4) 1 (0.6) – – – –	166 162 (97.6) 3 (1.8) 1 (0.6) – – –	
OAA/S T2, local anaesthetic injection, No (%) 5 awake 4 lethargic to normal tone 3 lethargic to loud tone 2 response mild prodding 1 response painful stimulus 0 no response painful stimulus	168 159 (94.6) 3 (1.8) 3 (1.8) – 3 (1.8) –	166 141 (84.9) 19 (11.4) 5 (3.0) – 1 (0.6) –	0.003
OAA/S T3, catheter tunnelling No (%) 5 awake 4 lethargic to normal tone 3 lethargic to loud tone 2 response mild prodding 1 response painful stimulus 0 no response painful stimulus	167 155 (92.8) 6 (3.6) 3 (1.8) – 2 (1.2) 1 (0.6)	166 121 (72.9) 33 (19.9) 9 (5.4) 1 (0.6) – 2 (1.2)	< 0.001
OAA/S T4, drape removal, No (%) 5 awake 4 lethargic to normal tone 3 lethargic to loud tone 2 response mild prodding 1 response painful stimulus 0 no response painful stimulus	165 163 (98.8) – 1 (0.6) – – 1 (0.6)	163 154 (94.5) 7 (4.3) 2 (1.2) – – –	
Procedure time min, median (IQR), [No.]	31.5 (25-40) [164]	30.0 (25-36) [165]	0.08
Postop observation time min, median (IQR), [No.]	17.0 (9-32) [167]	16.0 (9-35) [165]	

Abbreviations: No, Number; n, sample size; LA, local anaesthetic; PCS, patient-controlled sedation SVP, subcutaneous venous port; Fr, French; ml, millilitre; mg, milligram; OAA/S, Observer’s Assessment of Alertness/Sedation score; min, minutes

Footnote: ^a^: documentation on one antibiotic type was missing.

### Primary outcome

Global QoR-15 scores and domain scores are presented in Fig 2 and Table 3 (S4 Table), respectively. Preoperatively, the mean QoR-15 score was 122.4 (19.3) and 119.9 (21.6) in the LA group and LA + PCS group, respectively. There was no overall difference in total QoR-15 score between treatment groups (P = 0.61). Between-group comparisons showed no significant differences either postoperatively before discharge (mean difference 1.9; 95% CI −0.6–4.4; P = 0.14) or on postoperative day 1 (mean difference −2.4; 95% CI −5.1–0.2; P = 0.07). (S5 Table)

**Fig 2:**
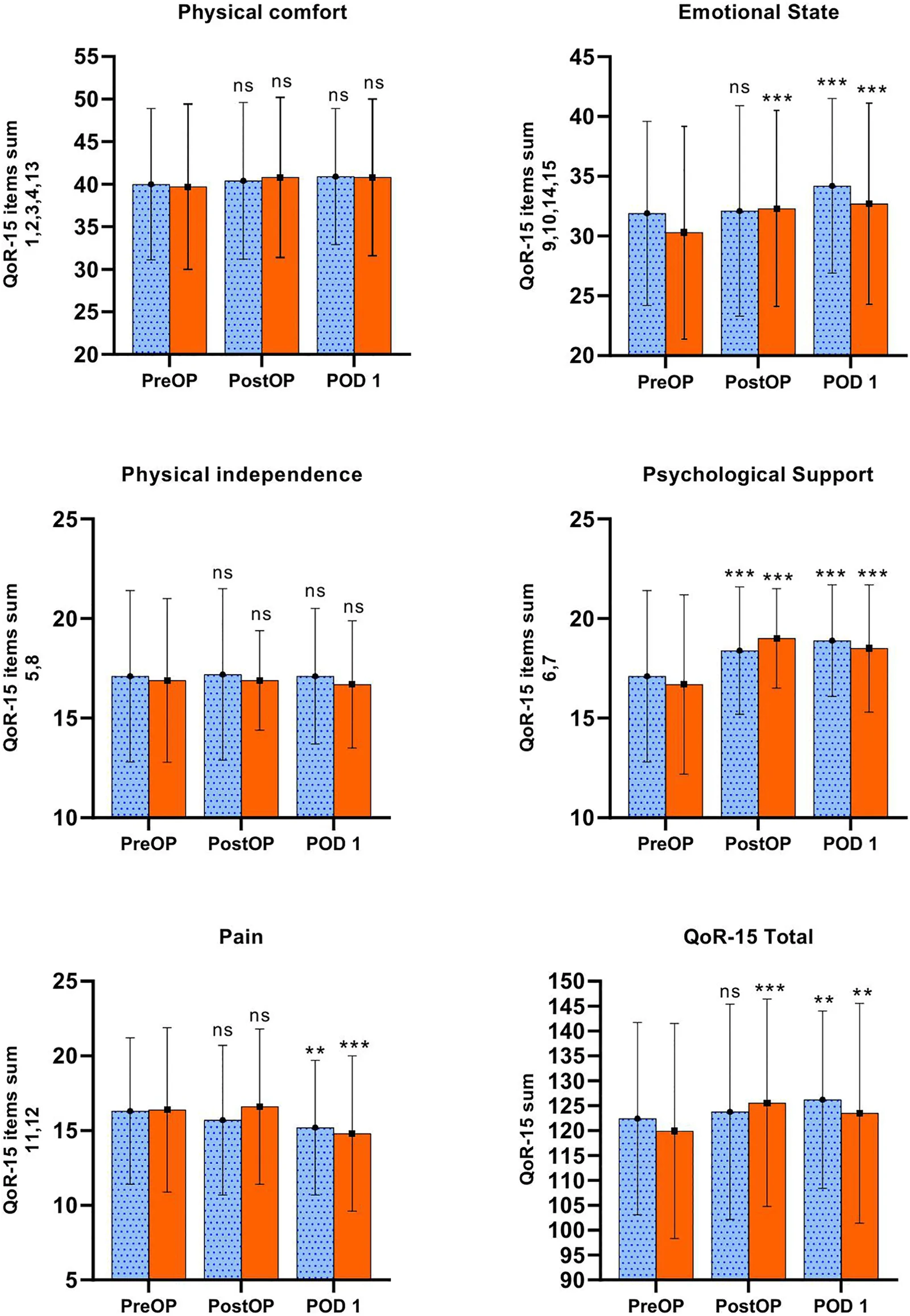
Quality of Recovery-15 (QoR-15) total and domain scores before and after venous port implantation. LA group; LA + PCS group. Abbreviations: PreOP, preoperative; PostOP, postoperatively; POD1,postoperative day 1; LA, local anaesthesia; PCS, patient-controlled sedation. Mean ± SD, P-values represent pairwise comparisons of postoperative and POD1 values with the corresponding preoperative baseline within each group. NS, not significant; **, p = 0.05 - ≤ 0.001; ***, p < 0.001.

### Secondary outcomes

Total QoR-15 scores changed significantly over time (P < 0.001), and the pattern of change over time differed between the treatment groups (group-by-time interaction, P = 0.02). Compared to baseline, mean QoR-15 scores increased to 126.2 (17.8) in the LA group (mean difference 2.9; 95% CI 0.4–5.5; P = 0.02) and to 123.5 (22.1) in the LA + PCS group (mean difference 3.1; 95% CI 0.5–5.6; P = 0.02) on POD 1. Although statistically significant, these changes did not exceed the published MCID of 6. The LA + PCS group reached its highest mean QoR-15 score postoperatively before discharge and showed a different pattern of change over time, with a mean increase of 5.6 points (95% CI 3.1–8.1; P < 0.001) compared with baseline.

The “Emotional state” and “Psychological support” domains changed significantly over time (P < 0.001 respectively). In addition, the pattern of change over time differed between treatment groups (group-by-time interaction: P < 0.05 and P = 0.02, respectively), with both domains reaching their highest scores before discharge in the LA + PCS group but not in the LA group. The individual QoR-15 item scores are presented in Table 4 (S6 Table). The pattern of change over time differed between the groups for the items “Getting support from hospital staff” (Psychological support), “Feeling sad or depressed” (Emotional state), and “Ability to breathe easily” (Physical comfort) (P < 0.05, P < 0.05, and P = 0.007, respectively).

The “Pain” domain was the only QoR-15 domain that showed a significant decline from baseline in both treatment groups on POD 1. The LA group declined to 15.2 (4.5) (mean difference −1.2 [95% CI −2.0- −0.4], P = 0.004) and the LA + PCS group to 14.8 (5.2) (mean difference −1.5 [95% CI-2.3- −0.6], P < 0.001) compared to preoperative values (S4 Table). Maximum pain on POD 1 was rated to a median of 3 (IQR 2–5) on NRS in both groups (P = 0.86) and a similar proportion of patients experienced maximum pain of ≥4 on NRS [74 patients (49.0%) LA group and 71 (48.0%) LA + PCS group; OR 1.042, 95% CI 0.66–1.64, P = 0.86].

The majority of patients took additional pain medication; 98 patients (64.9%) in the LA group and 108 patients (73%) in the LA + PCS group with paracetamol being the most commonly used analgesic in both groups [LA: 79 (80.6%); LA + PCS: 87 (80.5%)] (Table 3).

**Table 3:** Patients´ pain score on POD 1.

	LA (n = 168)	LA + PCS (n = 166)	P-value
Available data No (%) Maximum pain NRS after coming home median (IQR)	151 3 (2-5)	148 3 (2-5)	P = 0.86
Maximum pain after coming home NRS ≤ 3 NRS ≥ 4	77 (51.0) 74 (49.0)	77 (52.0) 71 (48.0)	P = 0.86
Taken additional pain medication? No Yes	53 (35.1) 98 (64.9)	40 (27.0) 108 (73.0)	P = 0.13
Medication taken? Paracetamol Ibuprofen Oxycodone/Morphine Other	79 (80.6) 4 (4.1) 9 (9.2) 6 (6.1)	87 (80.5) 4 (3.7) 13 (12.0) 4 (3.7)	
Would you have wanted better pain medication? No Yes	84 (85.7) 14 (14.3)	86 (79.6) 22 (20.4)	P = 0.25

Abbreviations: IQR, interquartile range; LA, local anaesthetic; PCS, patient-controlled sedation; No, Number; n, sample size.

## Discussion

We found no overall difference in postoperative total QoR-15 scores between patients receiving local anaesthesia alone and those receiving local anaesthesia combined with patient-controlled sedation. However, patients in the LA + PCS group showed a different pattern of change over time, reaching their highest QoR-15 score before discharge. This finding was primarily driven by the “Emotional state” and “Psychological support” domains. Although total QoR-15 scores improved from baseline in both groups by POD 1, these improvements did not exceed the published MCID of 6. The “Pain” domain was the only QoR-15 domain that declined significantly from baseline to POD 1 in both groups. Despite a median NRS pain score of 3, approximately half of the patients reported moderate-to-severe pain (NRS ≥ 4), indicating that postoperative pain remained a clinically relevant concern after SVP implantation.

Previous research on patients´ experiences during SVP implantation has primarily focused on intraoperative outcomes, including pain perception [16,17], patient satisfaction [18], and anxiety [19], to evaluate the effectiveness of various analgesic and sedative strategies. While important, these outcomes reflect only one dimension of the perioperative experience [20], may overlook broader aspects of recovery and neglect the postoperative period.

The QoR-15 questionnaire is a validated questionnaire commonly used in the perioperative setting and is recommended as an outcome measure by the ESA-ESICM joint task force on perioperative outcome measures [21]. To date, postoperative recovery after SVP implantation has not been specifically examined. QoR15 has been used as an outcome measure in a broad range of studies [22] including several RCT`s comparing different anaesthetic and analgesic strategies across various surgical settings such as urologic, laparoscopic, and thoracoscopic procedures [23–27]. Additionally, an observational study assessing QoR-15 with PCS in patients undergoing gastrointestinal endoscopic procedures demonstrated improved recovery scores on POD 1 [28]. Comparisons across studies are challenging owing to substantial heterogeneity in surgical procedures, patient populations, pharmacological regimens, and sedation protocols.

The different trajectory of recovery observed in the PCS group is likely related to the pharmacological effects of the medication used. Propofol has been reported to alleviate anxiety-like behaviours [29] and induce positive mood [30] as well as euphoric effects [31] when administered for clinical sedation. These psychopharmacological properties may partly explain the transient improvement observed in the emotional and psychological QoR-15 domains before discharge. These findings are particularly interesting in the context of PACSPI 2, which identified no between-group differences in intraoperative pain perception but did not assess other factors such as anxiolysis, emotional comfort or recall, emphasising the importance of more complex outcome measures [13].

All participating patients had recently been diagnosed with cancer with varying treatment intentions. Psychological distress is known to peak at the time of diagnosis [32] and a cancer diagnosis is widely recognized as a profoundly distressing and potentially traumatic experience for patients [33–36]. The low scores observed on relevant QoR-15 items suggest that patients who arrived for the procedure felt fatigued, experienced poor sleep, diminished well-being, and uncertainty regarding support from hospital staff, reflecting a preoperative state of distress. Although sleep quality and rest did not improve on POD 1 in either group, patients reported an increased sense of support from hospital staff and an improved sense of general well-being. These findings may be attributed to relief following the completion of the procedure and the development of a supportive relationship with the healthcare team. The importance of the patient-healthcare professional relationship in cancer care should not be underestimated, as it plays a crucial role in patient coping [37]. Previous research has highlighted its influence on recovery, health outcomes [38] and patient satisfaction, including in the context of SVP insertion [19].

Depression, anxiety and psychological distress have been associated with higher pain scores in cancer patients [39]. Despite a median NRS pain score of 3 on POD 1 in both groups, nearly half of the patients reported experiencing substantial pain (NRS ≥ 4) on POD 1. Notably, “Moderate pain” was the only QoR-15 item that declined from baseline in both groups. Pain intensity on POD 1 has been assessed following a wide range of surgical procedures and the pain levels reported in our trial are comparable to NRS scores observed after interventions such as toe amputation, breast quadrant resection or carotid endarterectomy [40]. While focusing on optimising intraoperative pain management, pain experienced on POD 1 may be underestimated due to limited follow-up, unclear patient instructions or patients´ concerns regarding potential side effects of analgesic medication [41]. These findings suggest that postoperative pain after SVP implantation deserves greater attention and support the need for improved patient education, realistic postoperative expectations, and effective postoperative analgesia. Given that opioid use after minor surgical procedures has not been shown to significantly reduce pain but is associated with an increased risk of adverse effects [42], alternative opioid-free analgesic strategies should be explored.

The strengths of this prespecified substudy include the use of a validated, patient-reported outcome instrument capable of capturing the multidimensional aspects of perioperative recovery of cancer patients, together with the robust sample size and a pragmatic multicentre design of the parent trial PACSPI-2, which enhances external validity.

Nevertheless, this study has several limitations. Approximately one-third of patients who declined to participate did so because they wished to receive sedation. These decisions may have been driven by procedural anxiety, fear of pain, or prior adverse experiences. Consequently, the study population may not fully represent patients most likely to benefit from PCS, introducing the possibility of selection bias despite randomisation and limiting generalisability. Furthermore, the lack of blinding may have introduced bias in the assessment of subjective patient-reported outcomes, as participants’ awareness of treatment allocation could have influenced their perceptions of treatment benefit. In addition, repeated QoR-15 analyses were based on available outcome data. However, participants with incomplete follow-up on POD 1 had slightly lower baseline QoR-15 scores, although no differences were observed in age, sex, BMI or treatment allocation. The complete-case sensitivity analysis yielded findings consistent with the primary analysis. Finally, the analyses of individual QoR-15 domains and items were exploratory and involved multiple statistical comparisons. Consequently, some statistically significant findings may represent chance findings and should be interpreted cautiously. Emphasis should therefore be placed on the overall pattern and consistency of the results rather than on individual P-values.

## Conclusion

Early postoperative recovery after SVP implantation was generally good and did not differ between the LA and LA + PCS group. A transient within-group improvement in emotional and psychological recovery domains was observed before discharge in the LA + PCS group. Pain on POD 1 remained common in both groups. As these findings were derived from prespecified secondary analyses without adjustment for multiple comparisons, they should be interpreted as exploratory.

## Supporting information

S1 Text CONSORT_Checklist. (DOC)

S1 File Statistical Analysis Plan (PACSPI 2). (DOCX)

S2 File Study Protocol (Secondary QoR-15 analysis). (DOCX)

S3 File Sensitivity analyses and comparison of participants with complete and incomplete QoR-15 follow-up. (DOCX)

S4 Table QoR-15 domain scores. (DOCX)

S5 Table Between-group differences in QoR-15 scores. (DOCX)

S6 Table QoR-15 item scores. (DOCX)
