# Supplementary material for: Quality of recovery (QoR-15) after patient-controlled sedation during venous port implantation: A prespecified substudy of the PACSPI-2 randomised clinical trial
Source: PLoS One. 2026 Oct 14;21(10):e0360479. doi: 10.1371/journal.pone.0360479 (PMC13667803; doi:10.1371/journal.pone.0360479)
Supplement: Supplementary file 2 [file pone.0360479.s003.docx]

Study protocol PACSPI 3

| TRIAL FULL TITLE | Patient-controlled Sedation in Port Implantation (PACSPI_3) – patient reported outcome measure on QoR-15 |
| --- | --- |
| EPM Nr  EPM amendment Nr | EPM: 2022-00088-01  EPM: 2022-00088-02 |
| EUDRACT NUMBER | 2021-003821-31 |
| PROTOCOL VERSION | 3.0 |
| STUDY CODE | Futurum-963747 |
| VERSION DATE | 2024-05-22 |
| TRIAL STATISTICIAN | Olle Eriksson |
| TRIAL SPONSOR | Knut Taxbro |
| TRIAL PRINCIPAL INVESTIGATOR | Stefanie Seifert |
| PROTOCOL AUTHOR | Stefanie Seifert |

Trial Steering Group

Trial Sponsor

Knut Taxbro

OP/IVA kliniken

Ryhov County Hospital

55185 Jönköping

Email: knut.taxbro@rjl.se>

Prinicipal Investigator

Stefanie Seifert

OP/IVA kliniken

Ryhov County Hospital

55185 Jönköping

Email: stefanie.seifert@rjl.se>

Co-Investigator

Fredrik Hammarskjöld

OP/IVA kliniken

Ryhov County Hospital

55185 Jönköping

Email: fredrik.hammarskjold@rjl.se>

Co-Investigator

Josip Azman

An/OP/IVA kliniken

Linköping University Hospital

Email: Josip.Azman@regionostergotland.se>

Co-Investigator

Andreas Nilsson

An/OP/IVA kliniken

Linköping University Hospital

Email: Andreas.Nilsson@regionostergotland.se>

Co-Investigator

Michelle Chew

An/OP/IVA kliniken

Linköping University Hospital

Email: Michelle.Chew@regionostergotland.se>

Statistician

Olle Eriksson

Futurum

Ryhov County Hospital

55185 Jönköping

Email: olle.eriksson@rjl.se>

Monitor:

FORUM Östergötland

Region Östergötland

Universitetssjukhuset I Linköping

58185 Linköping

forumo@regionostergotland.se

Table of Contents

[1	Abbreviations and Definitions	4](#_Toc174452735)

[2	Introduction	5](#_Toc174452736)

[2.1	Purpose of the analyses	6](#_Toc174452737)

[3	Study Objectives and Endpoints	6](#_Toc174452738)

[3.1	Study Objectives	6](#_Toc174452739)

[3.2	Endpoints	6](#_Toc174452740)

[4	Study Methods	7](#_Toc174452741)

[4.1	General Study Design and Plan	7](#_Toc174452742)

[4.2	Inclusion-Exclusion Criteria and General Study Population	7](#_Toc174452743)

[4.3	Randomisation and Blinding	7](#_Toc174452744)

[4.4	Study Variables	8](#_Toc174452745)

[5	Sample Size	9](#_Toc174452746)

[6	General Considerations	9](#_Toc174452747)

[6.1	Timing of Analyses	9](#_Toc174452748)

[6.2	Analysis Populations	9](#_Toc174452749)

[6.2.1	Full Analysis Population	9](#_Toc174452750)

[6.2.2	Per Protocol Population	9](#_Toc174452751)

[6.2.3	Intention to treat Population	10](#_Toc174452752)

[6.3	Missing Data	10](#_Toc174452753)

[6.4	Interim Analyses and Data Monitoring	10](#_Toc174452754)

[6.4.1	Documentation of Interim Analyses	10](#_Toc174452755)

[6.5	Multi-centre Studies	10](#_Toc174452756)

[6.6	Demographic and Baseline Variables	10](#_Toc174452757)

[7	Statistical Analyses	11](#_Toc174452758)

[8	Reporting Conventions	11](#_Toc174452759)

[9	Technical Details	11](#_Toc174452760)

# 1 Abbreviations and Definitions

| AE | Adverse Event |
| --- | --- |
| ASA | American Society of Anesthesiologists |
| CCS | Clinician-controlled sedation |
| CONSORT | Consolidated Standards of Reporting Trials |
| CRF | Case Report Form |
| DSMB | Data and Safety Monitoring Board |
| EPM | Etikprövningsmyndigheten, Swedish Ethical Review Authority. |
| GCP | Good Clinical Practice |
| ITT | Intention-to-treat |
| LA | Local Anaesthesia |
| LMV | Läkemedelsverket, Swedish medical products agency |
| MCID | Minimal clinically important difference |
| NRS | Numeric Rating Scale |
| PACU | Post Anesthesia Care Unit |
| PCS | Patient-controlled sedation |
| QoR-15 swe | Quality of Recovery-15 swedish |
| RCT | Randomized Controlled Trial |
| SFAI | Swedish Society for Anaesthesia and Intensive Care |
| SPOR | Swedish Perioperative Register |
| SVP | Subcutaneous Venous Port |
| TIVAD | Totally Implantable Venous Access Device |

# 2 Introduction

Cancer requiring chemotherapy is common. Approximately 70.000 patients are diagnosed with cancer in Sweden every year often leaving patients in need of intravenous chemotherapy. Subcutaneous venous ports (SVP) are commonly used in cancer patients to facilitate intravenous administration of chemotherapy, blood sampling and administration of other medications.

The implantation of SVPs is a minor surgical procedure and one of Sweden´s most common according to the Swedish Perioperative Register (SPOR). SVP implantation is usually performed in a day case surgery setting and the patient released home within a few hours after the procedure.

Conventional outcomes measured after surgery have focused on morbidity, mortality, pain scores, analgesic consumption, etc. However, these measures represent only one aspect of a patient`s recovery [1].

`Quality of Recovery` scores are patient-reported outcome measures (PROM) evaluating recovery after surgery and anaesthesia reflecting the patient`s ability to resume normal activities and are an important indicator of a successful perioperative experience [1].

The Quality of Recovery-15 (QoR-15) is a psychometrically evaluated instrument with high reliability and validity, high responsiveness and good return rates in the postoperative setting [2]. It provides a meaningful overall evaluation of a patient´s recovery after surgery [3]. Fifteen questions assess five domains of patient-reported health status: pain, physical comfort, physical independence, psychological support and emotional state. The 11-point numerical rating scale leads to a minimum score of 0 (very poor recovery) and a maximum score of 150 (excellent recovery). QoR-15 scores negatively correlate with duration of surgery and analgesic usage. Its minimal clinically important difference (MCID) describes the smallest change in score that constitutes a meaningful change in health status and is recommended to be a value of six [4]. The Swedish version of QoR-15swe has recently been validated [5].

A common perception of postoperative recovery in cancer patients undergoing SVP implantation is that recovery is good. We hypothesize that postoperative QoR-15 score is unaffected among patients undergoing SVP implantation, randomized to PCS. To our knowledge no study has investigated postoperative recovery after SVP implantation in general and the influence of PCS in particular.

The aim of this pre-planned sub-study of the PACSPI trial (PACSPI-2, EUDRACT NUMBER 2021-003821-31) is to assess postoperative recovery, measured as QoR-15 among patients randomized to patient-controlled sedation (PCS) with alfentanil and propofol as adjunct to local anaesthesia (LA) during SVP implantation or standard care with LA. Patients participating in the study are interviewed with QoR-15 at three different time points; preoperatively (baseline), postoperatively at the post anaesthesia care unit (PACU, before discharge) and 24 hours postoperatively (by telephone).

## 2.1 Purpose of the analyses

The analyses will assess recovery scores measured by QoR-15. The analyses will assess if there are differences in QoR-15 scores due to PCS intervention.

# 3 Study Objectives and Endpoints

## 3.1 Study Objectives

This study measures postoperative outcome of cancer patients undergoing SVP implantation evaluated with QoR-15 and the influence of PCS on postoperative recovery. The study was pre-planned within `Patient-controlled sedation in subcutaneous port implantation- a randomized controlled trial` (PACSPI-2, EUDRACT NUMBER 2021-003821-31) which assesses the efficacy of patient-controlled sedation (PCS) with alfentanil and propofol as adjunct to local anaesthesia during SVP implantation. Patients participating in the study are interviewed with QoR-15 at three different time points; preoperatively (baseline), postoperative in PACU (before discharge) and 24 hours postoperatively (by telephone). Assessment of QoR-15 scores between groups and time points are the study objectives in these analyses.

## 3.2 Endpoints

Primary endpoint:

The primary endpoint is assessment of change in total QoR-15 score between groups at the time points.

Secondary endpoints:

Secondary endpoint is assessment of difference for each QoR dimension and for each of Parts A and B within and between groups.

Secondary endpoint is assessment of analgesic consumption and patient-reported postoperative pain perception between groups.

# 4 Study Methods

## 4.1 General Study Design and Plan

This is a pre-planned secondary study of the PACSPI-2 trial (NCT05688384).

In short:

The PACSPI-2 trial is an open multicentre randomized controlled trial with a study and a control arm in a 1:1 ratio. Patients are randomized to either a control arm of LA for SVP-implantation or a study arm of PCS with propofol and alfentanil as adjunct to LA for SVP-implantation. The aim is to randomize 340 patients with an estimated patient recruitment over 18 months. The trial is performed at two centres; Ryhov County Hospital, Jönköping, Sweden and Linköping University Hospital, Sweden. QoR-15 data is collected during the PACSPI-2 trial. Secondary outcomes include patient satisfaction, implantation conditions, sedation level, sedative and analgesic medication consumption, postoperative analgesic requirement, procedural time consumption as well as safety aspects and adverse events.

## 4.2 Inclusion-Exclusion Criteria and General Study Population

Inclusion criteria:

- Adult patients (≥18 years) with cancer scheduled for SVP-implantation at participating anesthesia departments.

Exclusion criteria:

- Inability to operate the PCS apparatus
- Inability to communicate in Scandinavian languages
- Patients who require general anesthesia or patients eligible for LA only on anesthesiologist´s assessment (i.e. severe sleep apnea)
- Propofol or alfentanil allergy
- Non-fasting according to guidelines of the Swedish Society for Anesthesia and Intensive Care (SFAI)
- Failure to achieve peripheral vascular access
- Pregnancy
- Previous participation in study

## 4.3 Randomisation and Blinding

Prior to SVP-implantation and when the patient´s eligibility has been confirmed and consent forms have been completed the patient is randomized by password-coded randomization software accessible to the co-investigators at each participating site (REDCap). All patients are randomized onto the study prior to SVP-implantation.

Each patient randomized will be allocated a unique sequential patient code number for the randomization arm together with an allocated study arm.

The randomization scheme is equal allocation (1:1). Randomization will be performed consecutively. If a patient withdraws from the trial the unique patient code will not be used again and the patient will not be included into the trial again.

## 4.4 Study Variables

All measured study variables are described in the PACSPI-2 study protocol accessible on clinicaltrials.gov (NCT05688384).

QoR-15swe (Quality of Recovery-15 Sweden) is measured on a numeric rating scale (NRS) from 0 to 10.

The score in the separate dimensions will be combined into a single value as expression for total QoR-15 score.

Del A

Hur har du mått de senaste 24 timmarna?

På en skala från 0 till 10, där 0 = Inte någon gång {dåligt} och 10 = hela tiden {utmärkt}

| 1 | Kunnat andas lugnt | Inte någon gång | 0 | 1 | 2 | 3 | 4 | 5 | 6 | 7 | 8 | 9 | 10 | hela tiden |
| --- | --- | --- | --- | --- | --- | --- | --- | --- | --- | --- | --- | --- | --- | --- |
| 2 | Kunnat njuta av maten | Inte någon gång | 0 | 1 | 2 | 3 | 4 | 5 | 6 | 7 | 8 | 9 | 10 | hela tiden |
| 3 | Känt dig utvilad | Inte någon gång | 0 | 1 | 2 | 3 | 4 | 5 | 6 | 7 | 8 | 9 | 10 | hela tiden |
| 4 | Kunnat sova gott | Inte någon gång | 0 | 1 | 2 | 3 | 4 | 5 | 6 | 7 | 8 | 9 | 10 | hela tiden |
| 5 | Kunnat sköta toalettbesök och personlig hygien utan hjälp | Inte någon gång | 0 | 1 | 2 | 3 | 4 | 5 | 6 | 7 | 8 | 9 | 10 | hela tiden |
| 6 | Kunnat kommunicera med anhöriga eller vänner | Inte någon gång | 0 | 1 | 2 | 3 | 4 | 5 | 6 | 7 | 8 | 9 | 10 | hela tiden |
| 7 | Fått stöd från sjukhuspersonal | Inte någon gång | 0 | 1 | 2 | 3 | 4 | 5 | 6 | 7 | 8 | 9 | 10 | hela tiden |
| 8 | Kan du utföra ditt arbete eller dina vanliga aktiviteter hemma | Inte någon gång | 0 | 1 | 2 | 3 | 4 | 5 | 6 | 7 | 8 | 9 | 10 | hela tiden |
| 9 | Känt dig trygg och haft kontroll över din tillvaro | Inte någon gång | 0 | 1 | 2 | 3 | 4 | 5 | 6 | 7 | 8 | 9 | 10 | hela tiden |
| 10 | Haft en känsla av allmänt välbefinnande | Inte någon gång | 0 | 1 | 2 | 3 | 4 | 5 | 6 | 7 | 8 | 9 | 10 | hela tiden |

Del B

Har du känt något av följande symptom de senaste 24 timmarna?

på en skala från 10 till 0,där 10 = Inte någon gång {utmärkt} och 0 = hela tiden {dåligt}

| 11 | Medelsvår smärta | Inte någon gång | 10 | 9 | 8 | 7 | 6 | 5 | 4 | 3 | 2 | 1 | 0 | hela tiden |
| --- | --- | --- | --- | --- | --- | --- | --- | --- | --- | --- | --- | --- | --- | --- |
| 12 | Svår smärta | Inte någon gång | 10 | 9 | 8 | 7 | 6 | 5 | 4 | 3 | 2 | 1 | 0 | hela tiden |
| 13 | Illamående eller kräkning | Inte någon gång | 10 | 9 | 8 | 7 | 6 | 5 | 4 | 3 | 2 | 1 | 0 | hela tiden |
| 14 | Känt ångest eller oro | Inte någon gång | 10 | 9 | 8 | 7 | 6 | 5 | 4 | 3 | 2 | 1 | 0 | hela tiden |
| 15 | Känt dig ledsen eller deprimerad | Inte någon gång | 10 | 9 | 8 | 7 | 6 | 5 | 4 | 3 | 2 | 1 | 0 | hela tiden |

Time table for measurement of variables

|  | Preop  Baseline | Intraop | Postop  Before discharge | 24h postop  Telephone |
| --- | --- | --- | --- | --- |
| QoR-15swe | x |  | x | x |
| Demographic | x |  |  |  |
| Vital signs | x | x |  |  |
| Procedure details |  | x |  |  |
| Malignancy details | x |  |  |  |
| Sedation assessment |  | x |  |  |
| AE/SAE |  | x |  |  |
| Analgesia |  | x |  | x |
| Time consumption | x | x | x |  |

# 5 Sample Size

Sample size calculation in the original trial was performed on its primary endpoint with the aim to detect a 50% reduction in pain perception as stated by the study protocol. 340 patients are included in the study with a 1:1 randomization.

Considering a minimal clinically important difference (MCID) of 6 that has been suggested to constitute a meaningful change in health status with a standard deviation of 19 a total number of 314 patients is needed to detect this change with 80% power, 2-sided, at a significance level of 5%.

# 6 General Considerations

## 6.1 Timing of Analyses

The final analysis will be performed after 340 patients have been included.

## 6.2 Analysis Populations

### 6.2.1 Full Analysis Population

All participants who were randomized.

### 6.2.2 Per Protocol Population

All participants who received a SVP.

### 6.2.3 Intention to treat Population

Analyses will be performed on all participants in the original assigned randomized group.

## 6.3 Missing Data

Missing data are assumed of missing at random (MAR). Less than 10 % of data are assumed missing. Missing data will be quantified and reported.

## 6.4 Interim Analyses and Data Monitoring

Interim analyses are not expected to lead to early closure of neither randomization on safety nor efficacy grounds. Both techniques are established and clinically approved methods and complications are very uncommon. After 100 respectively 200 patients analyses of adverse events (AE) will be performed in which we expect a frequency lower than 15% based on previous studies (PACSPI1). In the event of a higher frequency the sponsor will discuss further action with the monitor.

### 6.4.1 Documentation of Interim Analyses

Snapshots of the data available at each interim analysis will be preserved. Reports of AE analyses will be stored in the sponsor`s documents enabling recreating the decision process from the trial archive.

## 6.5 Multi-centre Studies

This trial will be carried out in the following anaesthesia departments:

OP/IVA-klinken, Ryhov County Hospital, Sweden

AnOpIVA-kliniken, Linköping University Hospital, Sweden

Co-investigators at participating sites will have access to REDCap. Centres are provided with the PCS pump. There will be no restrictions in the choice of LA-solution being left up to local practice at participating sites

## 6.6 Demographic and Baseline Variables

Gender, age, American Society of Anestesiologists (ASA)-class, BMI, cancer type, treatment strategy, preoperative analgesia, procedural time, sedation score at timepoints T1-4 (OAAS), sedation volym, alfentanil (mg) delivered, propofol (mg) delivered will be reported for demographic and interventional data.

# 7 Statistical Analyses

The analysis will be produced on intention to treat population.

Data will be summarised by treatment group. Summary statistics of demographic and baseline variables will be reported. All continuous variables will be summarised using the following descriptive statistics: n (non-missing sample size), median, interquartile range. The frequency and percentages (based on the non-missing sample size) of observed levels will be reported for all categorical measures. Categorical data will be analysed with Chi2 test, continuous data will be analysed with Mann-Whitney U test.

Repeated measurement of analysis of variance is used for the analysis of treatment effect of PCS on QoR-15 scores. This model takes the inter- and intra-individual variation into account and provides flexibility in handling missing data which will be reported. Fixed factors include treatment group, time and treatment-by time-interaction. Confidence intervals will be reported and adjustment for planned comparison conducted. Testing will be two-sided with a 5% significance level.

Demographic and baseline data will be listed by treatment group in a table.

Primary endpoint will be displayed in a figure.

Secondary endpoints will be displayed in a figure.

# 8 Reporting Conventions

P-values ≥ 0.01 will be reported to two decimal places, p-values ≥0.001 will be reported to three decimal places; p-values less than 0.001 will be reported as “<0.001”. The median and any other statistics other than quantiles, will be reported to one decimal place greater than the original data.

# 9 Technical Details

Analyses will be carried out with the study statistician. Analyses will be carried out in SPPS, IBM

References

1.	Chazapis M, Walker EM, Rooms MA, Kamming D, Moonesinghe SR. (2016). Measuring quality of recovery-15 after day case surgery. Br J Anaesth;116(2):241-8. http://dx.doi.org/10.1093/bja/aev413.

2.	Stark PA, Myles PS, Burke JA. (2013). Development and psychometric evaluation of a postoperative quality of recovery score: the QoR-15. Anesthesiology;118(6):1332-40. http://dx.doi.org/10.1097/ALN.0b013e318289b84b.

3.	Myles PS. (2018). Measuring quality of recovery in perioperative clinical trials. Curr Opin Anaesthesiol;31(4):396-401. http://dx.doi.org/10.1097/aco.0000000000000612.

4.	Myles PS, Myles DB. (2021). An Updated Minimal Clinically Important Difference for the QoR-15 Scale. Anesthesiology;135(5):934-5. http://dx.doi.org/10.1097/aln.0000000000003977.

5.	Lyckner S, Böregård IL, Zetterlund EL, Chew MS. (2018). Validation of the Swedish version of Quality of Recovery score -15: a multicentre, cohort study. Acta Anaesthesiol Scand;62(7):893-902. http://dx.doi.org/10.1111/aas.13086.
