# Supplementary material for: Quality of recovery (QoR-15) after patient-controlled sedation during venous port implantation: A prespecified substudy of the PACSPI-2 randomised clinical trial
Source: PLoS One. 2026 Oct 14;21(10):e0360479. doi: 10.1371/journal.pone.0360479 (PMC13667803; doi:10.1371/journal.pone.0360479)
Supplement: Supplementary file 3 [file pone.0360479.s004.docx]

Baseline characteristics of participants with complete and incomplete QoR-15 follow-up.

|  | Complete-case  (n = 294) | Incomplete follow-up  (n = 40) | P value |
| --- | --- | --- | --- |
| Age, years | 67.3 ± 12.8 | 66.4 ± 11.0 | 0.436 |
| Female sex, n (%) | 138 (46.9) | 23 (57.5) | 0.278 |
| BMI, kg/m² | 25.6 ± 4.6 | 26.0 ± 5.3 | 0.829 |
| LA, n (%) | 149 (50.7) | 19 (47.5) | 0.835 |
| LA + PCS n (%) | 145 (49.3) | 21 (52.5) |  |
| Baseline QoR-15 score | 121.9 ± 20.3 | 115.1 ± 20.9 | 0.042 |

Complete-case participants had QoR-15 total scores available at all three predefined assessment time points (preoperative, postoperative, and postoperative day 1). Participants with incomplete follow-up had at least one missing QoR-15 total score. Of the 40 participants with incomplete follow-up, 32 had no QoR-15 assessment on postoperative day 1 because telephone follow-up could not be completed. The remaining eight participants had incomplete QoR-15 questionnaires at the preoperative or predischarge assessment, resulting in missing total QoR-15 scores. Review of these questionnaires indicated that the missing total scores were primarily attributable to one or a few missing questionnaire items, suggesting random missingness rather than systematic loss of data.

Sensitivity analysis using complete-case data.

| Analysis | Available-case analysis  (n = 334) | Complete-case analysis  (n = 294) |
| --- | --- | --- |
| Participants contributing to analysis | All randomised participants with available QoR-15 data | Participants with complete QoR-15 total scores at all three assessment time points |
| Time effect | P < 0.001 | P < 0.001 |
| Treatment effect | P = 0.61 | P = 0.59 |
| Treatment × time interaction | P = 0.020 | P = 0.016 |

The complete-case sensitivity analysis produced results consistent with the primary available-case analysis. Total QoR-15 scores changed significantly over time in both analyses, while no statistically significant overall treatment effect was observed. The significant treatment-by-time interaction remained, indicating that exclusion of participants with incomplete QoR-15 data did not materially influence the study conclusions.
