# Supplementary material for: Quality of recovery (QoR-15) after patient-controlled sedation during venous port implantation: A prespecified substudy of the PACSPI-2 randomised clinical trial
Source: PLoS One. 2026 Oct 14;21(10):e0360479. doi: 10.1371/journal.pone.0360479 (PMC13667803; doi:10.1371/journal.pone.0360479)
Supplement: Supplementary file 4 [file pone.0360479.s005.docx]

Table 3 Total QoR-15 and Domain Scores

|  | Timepoint | LA  Mean SD | Mean difference (95%CI) | P value | LA+PCS  Mean SD | Mean difference (95%CI) | P value | Group P value | Time  P value | Group* Time  P value |
| --- | --- | --- | --- | --- | --- | --- | --- | --- | --- | --- |
| QoR-15_total | PreOp | 122.4 (19.3) |  |  | 119.9 (21.6) |  |  | 0.61 | <0.001 | 0.02 |
|  | PostOp | 123.8 (21.6) | 1.2 (-1.3-3.6) | 0.36 | 125.6 (20.8) | 5.6 (3.1-8.1) | <0.001 |  |  |  |
|  | POD 1 | 126.2 (17.8) | 2.9 (0.4-5.5) | 0.02 | 123.5 (22.1) | 3.1 (0.5-5.6) | 0.02 |  |  |  |
| QoR items  1,2,3,4,13  Physical comfort | PreOp | 40.0 (8.9) |  |  | 39.7 (9.7) |  |  | 0.92 | 0.15 | 0.58 |
|  | PostOp | 40.4 (9.2) | 0.3 (-0.8- 1.4) | 0.60 | 40.8 (9.4) | 1.1 (-0.0 - 2.3) | 0.06 |  |  |  |
|  | POD 1 | 40.9 (8.0) | 0.5 (–0.6 - 1.6) | 0.39 | 40.8 (9.2) | 0.8 (0.4 – 2.0) | 0.18 |  |  |  |
| QoR items  9,10,14,15  Emotional  state | PreOp | 31.9 (7.7) |  |  | 30.3 (9.0) |  |  | 0.23 | <0.001 | <0.05 |
|  | PostOp | 32.1 (8.8) | 0.3 (-0.8- 1.3) | 0.65 | 32.3 (8.2) | 2.0 (0.9 – 3.0) | <0.001 |  |  |  |
|  | POD 1 | 34.2 (7.3) | 1.9 (0.8 – 3.0) | <0.001 | 32.7 (8.4) | 2.2 (1.1 – 3.3) | <0.001 |  |  |  |
| QoR items  5,8  Physical indepencence | PreOp | 17.1 (4.1) |  |  | 16.9 (4.1) |  |  | 0.47 | 0.95 | 0.99 |
|  | PostOp | 17.2 (4.3) | 0.0 (-0.5 – 0.5) | 0.97 | 16.9 (4.4) | 0.0 (-0.5 -0.6) | 0.90 |  |  |  |
|  | POD 1 | 17.1 (3.4) | -0.0 (-0.6 – 0.5) | 0.92 | 16.7 (4.3) | -0.1 (-0.6-0.5) | 0.83 |  |  |  |
| QoR items  6,7  Psychological  support | PreOp | 17.1 (4.3) |  |  | 16.7 (4.5) |  |  | 0.62 | <0.001 | 0.02 |
|  | PostOp | 18.4 (3.2) | 1.3 (0.7-1.8) | <0.001 | 19.0 (2.5) | 2.3 (1.6-2.9) | <0.001 |  |  |  |
|  | POD 1 | 18.9(2.8) | 1.7 (1.2-2.3) | <0.001 | 18.5 (3.2) | 1.6 (1.0-2.3) | <0.001 |  |  |  |
| QoR items  11,12  Pain | PreOp | 16.3 (4.9) |  |  | 16.4 (5.5) |  |  | 0.63 | <0.001 | 0.19 |
|  | PostOp | 15.7(5.0) | -0.6 (-1.4-0.2) | 0.15 | 16.6 (5.2) | 0.2 (-0.6 – 1.0) | 0.66 |  |  |  |
|  | POD 1 | 15.2(4.5) | -1.2 (-2.0 - -0.4) | 0.004 | 14.8 (5.2) | -1.5 (-2.3 - -0.6) | <0.001 |  |  |  |

Abbreviations: CI, conficedence intervall, LA, local anaesthetic; QoR, Quality of Recovery; PCS, patient controlled sedation; PreOp; preoperative; PostOp; postoperative; POD1; postoperative day 1; SD, standard deviation;

Mean differences are expressed as pairwise comparisons of postoperative or POD 1 values compared to baseline values within each group.
