# Supplementary material for: Quality of recovery (QoR-15) after patient-controlled sedation during venous port implantation: A prespecified substudy of the PACSPI-2 randomised clinical trial
Source: PLoS One. 2026 Oct 14;21(10):e0360479. doi: 10.1371/journal.pone.0360479 (PMC13667803; doi:10.1371/journal.pone.0360479)
Supplement: Supplementary file 5 [file pone.0360479.s006.docx]

## Between-group comparisons with mean differences (95% confidence intervals) for total QoR-15 score and QoR-15 domain scores.

| Outcome | Preoperative  mean difference (95% CI) | P-value | Postoperative  mean difference (95% CI) | P-value | POD1  mean difference (95% CI) | P-value |
| --- | --- | --- | --- | --- | --- | --- |
| Total QoR-15 score | −2.54 (−5.01 - −0.08) | 0.04 | 1.90 (−0.59 - 4.38) | 0.13 | −2.43 (−5.06 - 0.21) | 0.07 |
| Physical comfort | −0.30 (−1.40 - 0.81) | 0.60 | 0.53 (−0.58 - 1.65) | 0.35 | 0.02 (−1.17 - 1.21) | 0.97 |
| Physical independence | −0.29 (−0.81 - 0.23) | 0.27 | −0.27 (−0.78 - 0.25) | 0.31 | −0.32 (−0.88 - 0.23) | 0.25 |
| Psychological support | −0.44 (−1.02 - 0.15) | 0.14 | 0.54 (−0.05 - 1.12) | 0.07 | −0.55 (−1.17 - 0.08) | 0.09 |
| Emotional state | −1.63 (−2.66 - −0.59) | 0.002 | 0.10 (−0.94 - 1.14) | 0.85 | −1.35 (−2.46 - −0.23) | 0.02 |
| Pain | 0.06 (−0.74 - 0.86) | 0.88 | 0.82 (0.02 - 1.62) | 0.04 | −0.22 (−1.08 - 0.63) | 0.61 |

Mean differences represent LA+PCS − LA and are based on estimated marginal means obtained from the univariate general linear model. Positive values indicate higher QoR-15 scores in the LA+PCS group. Confidence intervals are 95%.
