# Supplementary material for: Quality of recovery (QoR-15) after patient-controlled sedation during venous port implantation: A prespecified substudy of the PACSPI-2 randomised clinical trial
Source: PLoS One. 2026 Oct 14;21(10):e0360479. doi: 10.1371/journal.pone.0360479 (PMC13667803; doi:10.1371/journal.pone.0360479)
Supplement: Supplementary file 6 [file pone.0360479.s007.docx]

Table 4: QoR-15 Item Scores

| Item | Timepoint | LA group  Mean (SD) | Mean difference (95%CI) | P-value | LA+PCS group  Mean (SD) | Mean difference (95%CI) | P-value | Group  (P-value) | Time  (P-value) | Group *Time  (P-value) | Domain |
| --- | --- | --- | --- | --- | --- | --- | --- | --- | --- | --- | --- |
| 1. Able to breathe easily | Preop  Postop  POD1 | 9.5 (1.3)  9.3 (1.5)  9.7 (0.9) | -0.2 (-0.4-0.1)  0.2 (-0.1-0.1) | 0.23  0.11 | 9.1 (2.0)  9.5 (1.3) *  9.4 (1.5) * | 0.4 (0.1-0.6)  0.3 (0.0-0.6) | 0.003  0.04 | 0.19 | 0.03 * | 0.007* | Physical comfort |
| 2. Been able to enjoy food | Preop  Postop  POD1 | 7.4 (3.6)  7.5 (3.6)  8.2 (3.0) * | 0.1 (-0.4-0.5)  0.7 (0.3-1.2) | 0.74  <0.001 | 7.8 (3.3)  7.6 (3.5)  8.1 (2.9) | -0.1 (-0.6-0.3)  0.2 (-0.3-0.6) | 0.52  0.48 | 0.73 | 0.004 * | 0.19 | Physical comfort |
| 3. Feeling rested | Preop  Postop  POD1 | 6.9 (3.1)  7.3 (3.2)  6.7 (3.2) | 0.3 (-0.1-0.8)  -0.4 (-0.8-0.1) | 0.15  0.14 | 6.8 (3.3)  7.4 (3.0) *  7.1 (3.0) | 0.6 (0.1-1.1)  0.2 (-0.3-0.7) | 0.01  0.39 | 0.63 | 0.003 * | 0.26 | Physical comfort |
| 4. Have had a good sleep | Preop  Postop  POD1 | 7.0 (3.2)  7.0 (3.3)  6.8 (3.1) | 0.1 (-0.4-0.5)  -0.3 (-0.7-0.2) | 0.78  0.31 | 7.1 (3.1)  7.2 (3.1)  7.2 (3.0) | 0.2 (-0.3-0.6)  0.1 (-0.4-0.5) | 0.53  0.85 | 0.67 | 0.72 | 0.39 | Physical comfort |
| 5. Able to look after personal toilet and hygiene unaided | Preop  Postop  POD1 | 9.6 (1.6)  9.4 (1.9)  9.7 (1.4) | -0.2 (-0.4-0.1)  0.1 (-0.1-0.4) | 0.16  0.27 | 9.5 (1.7)  9.5 (1.9)  9.5 (1.6) | -0.0 (-0.3-0.2)  0.1 (-0.2-0.3) | 0.72  0.58 | 0.54 | 0.06 | 0.53 | Physical independence |
| 6. Able to communicate with family or friends | Preop  Postop  POD1 | 9.9 (0.7)  9.8 (1.1)  9.9 (0.5) | -0.1 (-0.2-0.0)  0.0 (-0.1-0.2) | 0.14  0.66 | 9.8 (0.8)  9.9 (0.6)  9.8 (0.9) | 0.0 (-0.1-0.2)  0.0 (-0.2-0.1) | 0.66  0.84 | 0.89 | 0.65 | 0.18 | Psychological support |
| 7. Getting support from hospital doctors and nurses | Preop  Postop  POD1 | 7.3 (4.3)  8.6 (2.9) *  8.9 (2.8) * | 1.4 (0.8-2.0)  1.7 (1.1-2.3) | <0.001  <0.001 | 6.9 (4.4)  9.1 (2.4) *  8.7 (3.0) * | 2.3 (1.7-2.8)  1.6 (1.0-2.2) | <0.001  <0.001 | 0.65 | <0.001 * | 0.05 * | Psychological support |
| 8. Able to return to work or usual home activities | Preop  Postop  POD1 | 7.5 (3.4)  7.7 (3.1)  7.4 (2.9) | 0.2 (-0.3-0.6)  -0.2 (-0.6-0.3) | 0.42  0.47 | 7.4 (3.5)  7.4 (3.5)  7.2 (3.5) | 0.1 (-0.4-0.5)  -0.1 (-0.6-0.3) | 0.74  0.57 | 0.54 | 0.24 | 0.90 | Physical independence |
| 9. Feeling comfortable and in control | Preop  Postop  POD1 | 8.8 (2.1)  8.6 (2.3)  9.0 (2.1) | -0.2 (-0.5-0.1)  0.1 (-0.3-0.4) | 0.27  0.74 | 8.5 (2.4)  8.8 (2.1) *  8.9 (2.2) * | 0.3 (0.0-0.7)  0.4 (0.0-0.7) | 0.05  0.03 | 0.67 | 0.20 | 0.09 | Emotional state |
| 10. Having a feeling of general well-being | Preop  Postop  POD1 | 6.9 (3.0)  7.6 (2.8) *  7.4 (2.7) * | 0.7 (0.3-1.0)  0.5 (0.1-0.9) | <0.001  0.02 | 6.6 (3.1)  7.5 (2.8) *  7.2 (2.9) * | 0.8 (0.4-1.2)  0.5 (0.1-0.9) | <0.001  0.009 | 0.47 | <0.001 * | 0.83 | Emotional state |
| 11. Moderate pain | Preop  Postop  POD1 | 7.2 (3.4)  6.9 (3.2)  6.2 (3.0) * | -0.3 (-0.8-0.3)  -1.1 (-1.6- -0.5) | 0.29  <0.001 | 7.6 (3.5)  7.7 (3.2)  6.2 (3.1) * | 0.1 (-0.4-0.6)  -1.3 (-1.8- -0.7) | 0.74  <0.001 | 0.16 | <0.001 * | 0.33 | Pain |
| 12. Severe pain | Preop  Postop  POD1 | 9.1 (2.4)  8.8 (2.5)  8.9 (2.3) | -0.3 (-0.7-0.1)  -0.2 (-0.6-0.3) | 0.16  0.47 | 8.8 (2.6)  8.9 (2.6)  8.5 (2.6) | 0.1 (-0.3-0.5)  -0.3 (-0.7-0.2) | 0.73  0.25 | 0.43 | 0.41 | 0.27 | Pain |
| 13. Nausea or vomiting | Preop  Postop  POD1 | 9.2 (2.1)  9.1 (2.2)  9.4 (1.8) | -0.0 (-0.3-0.3)  0.2 (-0.2-0.5) | 0.94  0.31 | 8.9 (2.4)  9.0 (2.4)  9.0 (2.2) | 0.1 (-0.2-0.4)  0.1 (-0.2-0.4) | 0.63  0.42 | 0.20 | 0.40 | 0.86 | Physical comfort |
| 14. Feeling worried or anxious | Preop  Postop  POD1 | 7.9 (2.9)  7.8 (3.0)  8.9 (2.2) * | -0.1 (-0.5-0.4)  0.9 (0.4-1.3) | 0.78  <0.001 | 7.8 (2.9)  8.0 (2.8)  8.3 (2.8) * | 0.2 (-0.2-0.6)  0.5 (0.0-0.9) | 0.39  0.03 | 0.52 | <0.001 * | 0.14 | Emotional state |
| 15. Feeling sad or depressed | Preop  Postop  POD1 | 8.3 (2.8)  8.1 (3.0)  8.9 (2.4) * | -0.2(-0.6-0.3)  0.5 (0.1-1.0) | 0.43  0.02 | 7.4 (3.2)  8.0 (2.9) *  8.2 (2.8) * | 0.6 (0.2-1.1)  0.8 (0.4-1.2) | 0.003  <0.001 | 0.05 | <0.001 * | 0.028 * | Emotional state |
| Abbreviations: LA, local anaesthetic; QoR, Quality of Recovery; PCS, patient controlled sedation; preop; preoperative; postop; postoperative; POD1; postoperative day 1; SD, standard deviation  Mean differences are expressed as pairwise comparisons of postoperative or POD 1 values compared to baseline values within each group. A * is added for better readability to highlight significant values within group compared to baseline preoperatively. | | | | | | | | | | | |
