# Supplementary material for: Quality of recovery (QoR-15) after patient-controlled sedation during venous port implantation: A prespecified substudy of the PACSPI-2 randomised clinical trial
Source: PLoS One. 2026 Oct 14;21(10):e0360479. doi: 10.1371/journal.pone.0360479 (PMC13667803; doi:10.1371/journal.pone.0360479)
Supplement: Supplementary file 7 [file pone.0360479.s009.docx]

PONE-D-26-09449

Quality of recovery after patient-controlled sedation in venous port implantation – a randomised clinical trial (PACSPI 3)

PLOS One

Dear Dr. Seifert,

Thank you for submitting your manuscript to PLOS ONE. After careful consideration, we feel that it has merit but does not fully meet PLOS ONE’s publication criteria as it currently stands. Therefore, we invite you to submit a revised version of the manuscript that addresses the points raised during the review process.

Please submit your revised manuscript by Jul 25 2026 11:59PM. If you will need more time than this to complete your revisions, please reply to this message or contact the journal office at plosone@plos.org. When you're ready to submit your revision, log on to https://www.editorialmanager.com/pone/ and select the 'Submissions Needing Revision' folder to locate your manuscript file.

Please include the following items when submitting your revised manuscript:

•	A letter that responds to each point raised by the academic editor and reviewer(s). You should upload this letter as a separate file labeled 'Response to Reviewers'.

•	A marked-up copy of your manuscript that highlights changes made to the original version. You should upload this as a separate file labeled 'Revised Manuscript with Track Changes'.

•	An unmarked version of your revised paper without tracked changes. You should upload this as a separate file labeled 'Manuscript'.

If you would like to make changes to your financial disclosure, please include your updated statement in your cover letter. Guidelines for resubmitting your figure files are available below the reviewer comments at the end of this letter.

We look forward to receiving your revised manuscript.

Kind regards,

Carlos Alberto Antunes Viegas, DVM; MSc; PhD

Academic Editor

PLOS One

Journal Requirements:

When submitting your revision, we need you to address these additional requirements.

1. Please ensure that your manuscript meets PLOS ONE's style requirements, including those for file naming. The PLOS ONE style templates can be found at

https://journals.plos.org/plosone/s/file?id=wjVg/PLOSOne_formatting_sample_main_body.pdf and

https://journals.plos.org/plosone/s/file?id=ba62/PLOSOne_formatting_sample_title_authors_affiliations.pdf

*We revised the style accordingly*

2. We note that the grant information you provided in the ‘Funding Information’ and ‘Financial Disclosure’ sections do not match.

When you resubmit, please ensure that you provide the correct grant numbers for the awards you received for your study in the ‘Funding Information’ section.

*Thank you. We updated the manuscript accordingly.*

3. Thank you for stating the following financial disclosure: [Funding was obtained from: Futurum, Academy of Health and Care, Jönköping County, Sweden (Grant number: Futurum-963747)].

Please state what role the funders took in the study. If the funders had no role, please state: ""The funders had no role in study design, data collection and analysis, decision to publish, or preparation of the manuscript.""

If this statement is not correct you must amend it as needed.

Please include this amended Role of Funder statement in your cover letter; we will change the online submission form on your behalf.

*Thank you. We revised the financial disclosure and the role of the funder and included the amendment in the cover letter.*

4. We noted in your submission details that a portion of your manuscript may have been presented or published elsewhere. [The baseline characteristics reported here have been previously published in the primary report of the parent trial. Their inclusion in this manuscript reflects the fact that the present analysis constitutes a pre-specified sub-study.] Please clarify whether this [conference proceeding or publication] was peer-reviewed and formally published. If this work was previously peer-reviewed and published, in the cover letter please provide the reason that this work does not constitute dual publication and should be included in the current manuscript.

*The parent PACSPI-2 trial has been formally published in a peer-reviewed journal. The present manuscript does not constitute duplicate publication, as it reports a prespecified substudy and secondary analysis of a distinct, prespecified secondary outcome (postoperative recovery assessed using the QoR-15 questionnaire) that was not reported in the primary publication. The limited overlap in baseline characteristics and study methods is necessary to describe the study population and trial design and reflects that both manuscripts originate from the same randomized clinical trial.*

5. Thank you for stating the following in the Acknowledgments Section of your manuscript: [Assistance: We thank all staff involved in the PACSPI2 and PACSPI3 trials at Linköping University Hospital and Ryhov County Hospital for their invaluable contributions.

Financial Support and Sponsorship: This study was supported by grants from Futurum Academy of Health and Care, Jönköping County (Futurum-963747) Sweden.

Role of the Funder/Sponsor: The study protocol was approved by Futurum. Futurum was not involved in the study’s design, conduct, data collection, data management, analysis, or interpretation. It did not participate in the manuscript’s preparation, review, approval, or the decision to submit it for publication. All aspects of data collection, management, analysis, and manuscript preparation were independently handled by Dr. Seifert at the Department of Anaesthesia and Intensive Care Medicine, Ryhov County Hospital, Jönköping, Sweden]

We note that you have provided funding information that is not currently declared in your Funding Statement. However, funding information should not appear in the Acknowledgments section or other areas of your manuscript. We will only publish funding information present in the Funding Statement section of the online submission form.

Please remove any funding-related text from the manuscript and let us know how you would like to update your Funding Statement. Currently, your Funding Statement reads as follows: [Funding was obtained from: Futurum, Academy of Health and Care, Jönköping County, Sweden (Grant number: Futurum-963747)].

*We removed any funding-related text from the manuscript and updated the funding statement. Financial Support: Funding was obtained from Futurum Academy of Health and Care, Jönköping County (Futurum-963747) Sweden.*

Please include your amended statements within your cover letter; we will change the online submission form on your behalf.

*We included the following amended statements in the cover letter: This study was supported by Futurum – Academy of Health and Care, Region Jönköping County, Sweden (Grant No. Futurum-963747). The funder had no role in study design, data collection and analysis, decision to publish, or preparation of the manuscript.*

6. In the online submission form, you indicated that [Data can be obtained from the corresponding author,].

All PLOS journals now require all data underlying the findings described in their manuscript to be *freely available* to other researchers, either 1. In a *public repository*, 2. Within the manuscript itself, *or* 3. *Uploaded as supplementary information*.

This policy applies to all data except where public deposition would breach compliance with the protocol approved by your research ethics board. If your data cannot be made publicly available for ethical or legal reasons (e.g., public availability would compromise patient privacy), please explain your reasons on resubmission and your exemption request will be escalated for approval.

*Thank you for this clarification. The individual participant data cannot be made publicly available because unrestricted public data sharing was neither included in the approved study protocol nor covered by the participant information and informed consent. Public deposition of the dataset would therefore not be consistent with the ethical approval granted by the Swedish Ethical Review Authority. We respectfully request an exemption from the journal's open data policy on these grounds.*

7. When completing the data availability statement of the submission form, you indicated that you will make your data available on acceptance. We strongly recommend *all authors decide on a data sharing plan before acceptance*, as the process can be lengthy and hold up publication timelines. Please note that, though access restrictions are acceptable now, *your entire data will need to be made freely accessible if your manuscript is accepted for publication*. This policy applies to all data except where public deposition would breach compliance with the protocol approved by your research ethics board. If you are unable to adhere to our open data policy, please kindly revise your statement to explain your reasoning and we will seek the editor's input on an exemption. Please be assured that, once you have provided your new statement, the assessment of your exemption will not hold up the peer review process.

*The datasets generated and analysed during the current study are not publicly available because the participants did not provide consent for unrestricted public sharing of individual-level data, and such sharing is not covered by the ethical approval for the study. De-identified data may be made available for legitimate research purposes from the corresponding author upon reasonable request, provided that the requesting investigator has obtained approval from their institutional research ethics committee (or equivalent body, where required) under applicable data protection regulations.*

8. *Please include your tables as part of your main manuscript* and *remove the individual files*. Please note that *supplementary tables* (should remain/ be uploaded) as *separate ""supporting information"" files"""*

*We have removed the separate files and upploaded the supplementary tables as supporting information.*

9. Please *include captions for your Supporting Information files* at the *end of your manuscript*, and *update any in-text citations to match accordingly*. Please see our Supporting Information guidelines for more information: <http://journals.plos.org/plosone/s/supporting-information>.

*We included captions accordingly.*

10. If the reviewer comments include a recommendation to cite specific previously published works, please review and evaluate these publications to determine whether they are relevant and should be cited. There is no requirement to cite these works unless the editor has indicated otherwise.

*We were recommended two specific works which we chose to cite.*

Reviewers' comments:

Reviewer's Responses to Questions

Comments to the Author

1. Is the manuscript technically sound, and do the data support the conclusions?

The manuscript must describe a technically sound piece of scientific research with data that supports the conclusions. Experiments must have been conducted rigorously, with appropriate controls, replication, and sample sizes. The conclusions must be drawn appropriately based on the data presented.

Reviewer #1: Yes

Reviewer #2: Yes

Reviewer #3: Yes

Reviewer #4: No

________________________________________

2. Has the statistical analysis been performed appropriately and rigorously?

Reviewer #1: Yes

Reviewer #2: No

Reviewer #3: Yes

Reviewer #4: No

________________________________________

3. Have the authors made all data underlying the findings in their manuscript fully available?

The PLOS Data policy requires authors to make all data underlying the findings described in their manuscript fully available without restriction, with rare exception (please refer to the Data Availability Statement in the manuscript PDF file). The data should be provided as part of the manuscript or its supporting information, or deposited to a public repository. For example, in addition to summary statistics, the data points behind means, medians and variance measures should be available. If there are restrictions on publicly sharing data—e.g. participant privacy or use of data from a third party—those must be specified.

Reviewer #1: Yes

Reviewer #2: No

Reviewer #3: Yes

Reviewer #4: No

________________________________________

4. Is the manuscript presented in an intelligible fashion and written in standard English?

PLOS ONE does not copyedit accepted manuscripts, so the language in submitted articles must be clear, correct, and unambiguous. Any typographical or grammatical errors should be corrected at revision, so please note any specific errors here.

Reviewer #1: Yes

Reviewer #2: Yes

Reviewer #3: No

Reviewer #4: Yes

________________________________________

*Dear reviewers!*

*We sincerely appreciate the time and effort you have dedicated to reviewing our manuscript, as well as your thoughtful and constructive feedback. We have carefully considered each of your comments and have addressed them point by point in the responses below.*

**Reviewer #1**: This is a well-designed, adequately powered RCT evaluating the effect of patient-controlled sedation on postoperative recovery using the validated QoR-15 questionnaire. The study addresses an important clinical question in day-case oncology procedures. However, several issues should be addressed to improve clarity and interpretability.

1.The primary outcome (total QoR-15 score) showed no significant difference between groups (P = 0.61). However, the abstract and conclusion emphasize a “clinically meaningful improvement” in the LA+PCS group before discharge. This could mislead readers. The authors should clearly state that no overall difference was observed, and the subgroup finding is exploratory.

*Thank you for this comment. We agree and have revised the manuscript throughout to clarify that no overall difference in total QoR-15 score was observed between the treatment groups. The transient improvement before discharge in the LA+PCS group is now described as a finding from a prespecified secondary analysis and interpreted as exploratory ( page 3, line 50-54; page 23, line387-392).*

2.Multiple secondary outcomes, domains, and time points were compared without correction for multiplicity (e.g., Bonferroni or FDR). The authors should either adjust p-values or clearly label these analyses as exploratory.

*We agree that this required clarification. No adjustment for multiple comparisons was performed because these analyses were prespecified secondary and exploratory analyses. This has now been stated explicitly in the Statistical Analysis section, Abstract, Discussion and Limitations. (page 10 , line 227; page 23, line 390)*

3.Missing data on POD1 was 9.6% in both groups (16 patients each). The authors did not describe the mechanism of missingness or perform sensitivity analyses. Consideration of multiple imputation or complete-case sensitivity analysis is recommended.

*Thank you for this important comment. We have clarified how missing outcome data were handled in the Statistical Analysis section. Repeated QoR-15 measurements were analysed using a univariate general linear model. Participants contributed all available QoR-15 measurements, while observations with missing QoR-15 values were excluded from the repeated-measures analyses, resulting in available-case analyses within the intention-to-treat population.*

*Missing POD1 data occurred in 16 participants (9.6%) in each treatment group. In response to the reviewer's suggestion, we performed a complete-case sensitivity analysis including participants with complete QoR-15 total scores at all three assessment time points. The results were consistent with the primary available-case analysis, indicating that missing outcome data did not materially influence the study conclusions (S4 Table).*

*We also compared baseline characteristics between participants with complete and incomplete follow-up (S3 Table). Participants with incomplete follow-up had slightly lower baseline QoR-15 scores, whereas no differences were observed in age, sex, BMI, or treatment allocation. Given the modest amount of missing data, the balanced distribution between treatment groups, and the consistent findings in the complete-case sensitivity analysis, we considered additional multiple imputation unnecessary.*

4.The open-label design may have influenced patient-reported outcomes, especially subjective domains like emotional state and psychological support. The authors should discuss the potential magnitude and direction of this bias.

*We agree. This limitation has now been expanded in the Discussion. We explicitly acknowledge that the lack of blinding may have influenced subjective patient-reported outcomes, particularly emotional and psychological recovery domains. (page 23, line 375)*

5.Only 334 of 791 screened patients (42.2%) were enrolled. A large proportion (n=349) declined, often due to preference for sedation. This limits generalizability. The authors should compare characteristics of participants vs. decliners, or discuss implications.

*Unfortunately, no baseline characteristics were available for patients who declined participation. We have therefore expanded the Discussion to acknowledge that patients declining participation frequently preferred sedation and that this may have introduced selection bias and limited the generalisability of our findings. (page 22, line 370 )*

6.This is a valuable addition. I recommend including it in the citation as it directly supports the statement that QoR-15 has been used as an outcome measure in RCTs across various surgical settings (specifically orthopaedic/shoulder arthroscopy and thoracoscopic surgery). (PMID: 41939749, PMID: 41083890).

*Thank you for this helpful suggestion. Both recommended studies have now been cited. (page 28, line496)*

**Reviewer #2**: This randomized trial compared local anesthesia alone versus with patient controlled sedation (PCS) for venous port implantation in 334 cancer patients. Overall recovery (QoR 15 scores) was similar between groups, though PCS provided a short term improvement in emotional well being before discharge. By postoperative day 1, improvements were minimal and not clinically meaningful, and about half of patients reported moderate pain regardless of anesthetic approach.

*We appreciate the reviewer's summary of our findings*

Minor suggested revisions:

1- The sample size calculation is clearly presented (MCID=6, SD=19, 80% power, α=0.05); however, the specific statistical test or model used to derive this calculation is not stated. This should be explicitly clarified.

*Thank you for this comment. We have clarified both the rationale and the statistical basis of the sample size calculation. The sample size of the parent PACSPI-2 trial was determined by its primary endpoint of maximal intraprocedural pain. For the present prespecified secondary analysis, we evaluated whether the available sample size was sufficient to detect the published minimal clinically important difference (MCID) of 6 points in the QoR-15 score. This calculation was based on a two-sided comparison of two independent means (equivalent to an independent-samples t test), assuming an SD of 19, 80% power, and a two-sided significance level of 0.05.* *Under these assumptions, a total sample size of 314 participants was required to detect the predefined difference. The manuscript has been revised accordingly. (page 9, line 200)*

f

2- A large number of secondary outcomes, domain scores, and individual QoR 15 items are analyzed without adjustment for multiplicity. Either apply an appropriate correction or explicitly justify treating these analyses as exploratory.

*We agree that this required clarification. No adjustment for multiple comparisons was performed because the domain, item, and pairwise analyses were prespecified secondary analyses intended to be exploratory. This has now been stated explicitly in the Statistical Analysis section, Results, Discussion, and Conclusion. ( page 10 , line 227; page 23, line 390)*

3- The use of repeated measures ANOVA is appropriate, but additional details are needed regarding model assumptions (e.g., sphericity) and whether any corrections (e.g., Greenhouse–Geisser) were applied if assumptions were violated.

*Thank you for highlighting this point. We have revised the Statistical Analysis section to describe the analytical approach more accurately. Repeated QoR-15 measurements were analysed using a univariate general linear model with treatment group, time point, and the treatment group-by-time interaction included as fixed effects, while patient ID nested within treatment group was included as the random term. Because the analyses were not performed using a classical repeated-measures ANOVA, assumptions specific to that approach, including sphericity and Greenhouse–Geisser corrections, were not applicable. (page 10, line 212)*

4- Approximately 10% of patients had missing POD1 data. The manuscript should clarify how missing data were handled (e.g., complete case analysis vs. imputation) and the assumed missingness mechanism.

*Thank you for this suggestion. We performed a complete-case sensitivity analysis including participants with complete QoR-15 total scores at all three assessment time points. The results were consistent with the primary available-case analysis, indicating that missing outcome data did not materially influence the study conclusions. In addition, we compared baseline characteristics between participants with complete and incomplete follow-up. Although participants with incomplete follow-up had slightly lower baseline QoR-15 scores, no differences were observed in age, sex, BMI, or treatment allocation. These additional analyses have been added to the Supplementary Information (S3 and S4 Tables) and are referenced in the Results section. (page 10, line 226; page 17, line 257)*

5- While the statistical tests used are listed, it would improve clarity to explicitly map each test to the outcomes analyzed and to indicate whether assumptions underlying these tests were assessed.

*Thank you for this suggestion. We have revised the Statistical Analysis section to clarify the analytical approach. Mann–Whitney U tests were used for continuous between-group comparisons, Pearson's chi-square or Fisher's exact tests for categorical variables, and repeated QoR-15 measurements were analysed using the univariate general linear model described above. We have also clarified which outcomes were analysed using each of these methods. (page 10, line 207)*

6- Although some mean differences and confidence intervals are provided, more consistent reporting of effect sizes with confidence intervals across primary and key secondary outcomes would strengthen interpretability.

*We agree. To improve interpretability, we have added a supplementary table reporting between-group differences in estimated marginal means with corresponding 95% confidence intervals for the primary outcome (total QoR-15 score) and the key secondary outcomes (QoR-15 domain scores) at each assessment time point. These analyses complement the primary model-based results and provide the requested effect estimates without altering the interpretation of the study findings. (S5*

*)*

7- The manuscript references the MCID, but the distinction between statistical significance and clinical relevance could be more consistently emphasized, particularly for secondary outcomes.

*We agree and have revised the manuscript throughout to distinguish statistical significance from clinical importance. Results are now consistently interpreted in relation to the published MCID for the QoR-15 score.*

Reviewer #3: This is an interesting study addressing an important topic relating to procedural sedation/analgesia (v.s. none) in cancer patients undergoing venous port implantation. The underlying concept, choice of analgesic strategy, and study methodology, were appropriate.

*We thank the reviewer for the positive assessment of the study and for these constructive suggestions*

The manuscript was generally well-written. The phrases “effects of time”, and “group-by-time” interactions should be restated in plain language for the average reader.

*Thank you for this helpful suggestion. We have revised the Results section to describe these statistical findings in plain language while retaining the statistical terminology. Specifically, we now explain that the pattern of change over time differed between treatment groups when referring to the group-by-time interaction. (page 17, line 267)*

The findings are believable and could be used to inform future practice.

Abstract

Line 22: this opening sentence is very poor, so should be rewritten. Cancer patients don’t “recover” (i.e. get cured) after port insertion.

*We agree and rephrased the opening sentence. (page 3, .line 50)*

Line 36: clarify when the QoR-15 scores were done. Delete

*Thank you. We have revised the text accordingly.*

Page 4

Line 72: delete CVAD (acronym not needed)

*Thank you. We have revised the text accordingly.*

Line 77: delete “groups of”

*Thank you. We have revised the text accordingly.*

Lie 78: rewrite as, “The procedure is typically performed in an ambulatory setting, …”

*We rephrased accordingly. (page 5, line 85)*

Page 5

Line 103: Rephrase the hypothesis – “unaffected” is too vague (be specific).

*Thank you. We have rephrased the hypothesis for clarity. (page 6, line 111)*

Page 8

Line 176: clarify exactly what QoR-15 scores were used (when?)

*Thank you. We have rephrased for clarity. (page 9, line 191*

Line 184: this needs a reference (Myles et al Anesthesiology 2021)

*Thank you. We have added Myles et al Anesthesiology 2021 in this section. (page 9, line 202)*

Line 189: clarify, was the baseline QoR-15 score used in RMANOVA?

*Thank you for this comment. Yes. The baseline QoR-15 score was included as the preoperative assessment, together with the postoperative before-discharge and postoperative day 1 assessments, in the repeated-measures analysis. We have clarified this in the Statistical Analysis section by explicitly describing the analytical model and the three assessment time points included in the analysis. (page 10, line 212)*

Page 18

Line 260: begin with “We found that …”

*Thank you. We have revised accordingly. (page 20, line 301)*

Line 265: not relevance, but importance

*We rephrased this sectin of the manuscript. (page 20, line 306*

Page 19

Lines 287-293: this is repetitive (delete)

*We deleted this part.*

**Reviewer #4**: The authors have submitted an assessment of the quality of recovery after patient-controlled sedation in venous port implantation.

My primary concern is that quality of recovery was a SECONDARY endpoint in the PACSPI 2 clinical trial (see NCT05688384). This submission represents that quality of recovery is the primary endpoint. That is absolutely false.

*Thank you for this important comment. We agree that the wording in the original manuscript could have created the impression that this was an independent randomised clinical trial. This was not our intention. We have revised the title, abstract, methods, results, discussion, and conclusion to consistently describe this work as a prespecified secondary analysis of the PACSPI-2 randomised clinical trial. We now explicitly state that the parent trial was powered for maximal intraprocedural pain and that QoR-15 was a prespecified secondary outcome. (page 1, line 1; page 9, line 200)*

This is the primary outcome of the study as updated on 2024-11-12:

“Primary Outcome: Maximal intraprocedural pain level on numeric rating scale. NRS Participants assessment of maximal pain level experienced during the procedure using a numeric rating scale from 0-10 (0=no pain, 10=worst pain I know) on patient questionnaire.”

This is the power analysis paragraph from the statistical analysis plan:

“The minimum requirement is to demonstrate that the addition of PCS to LA reduces pain perception compared to SVP-implantation with LA only. Based on the assumption that 25% of patients experience pain ≥4 on NRS scale under SVP-implantation, we aim to detect at least a 50% reduction with PCS as adjunct. To detect this reduction with 80% power, 2-sided, significance level 5% requires 308 patients in total using 1:1 randomization. With an assumed drop-out rate of 10% we aim to include 170 patients per arm to show a difference between study arms.”

*We agree. The manuscript has been revised throughout to clearly distinguish between the primary endpoint of the parent PACSPI-2 trial and the primary outcome of the present prespecified secondary analysis. (page 2, line 31; page 9, line 200)*

Very clearly, the primary outcome was the difference in pain scores. It is false to claim that the primary outcome was quality of recover.

AFTER THE STUDY WAS COMPLETED, THE AUTHOR POSTED A NEW PROTOCOL SPECIFYING THAT THE OUTCOME WAS QUALITY OF RECOVERY.

*Thank you for raising this concern. We have clarified the chronology in the revised manuscript. QoR-15 was a prespecified secondary outcome of the PACSPI-2 trial. The PACSPI-3 protocol did not introduce a new study or new outcome but prespecified the statistical analysis of this secondary outcome before completion of the trial, database lock, and analysis of the study data. This chronology can also be verified in the ClinicalTrials.gov registry history. We have clarified this relationship throughout the manuscript.*

After completing the study, the authors revised the power analysis and sample size calculation to represent that this study, PACSPI-3, is a new-study following PACSPI-3. However, it isn’t. It’s the same patients in the already published PACSPI-2 study, with exactly the same Consort diagram. The authors correctly report the primary outcome in that study as “The primary outcome was maximum pain score during the procedure, assessed using the 11‐point NRS in the recovery unit before discharge (0 = no pain; 10 = worst pain imaginable).”

The authors describe a power analysis for the Quality of Recovery endpoint, claiming that this determined the sample size. That’s just false. As stated above, the power analysis was determined for pain scores.

*We agree and appreciate this clarification. The sample size of the parent PACSPI-2 trial was determined by the primary endpoint of maximal intraprocedural pain. We have revised the Statistical Analysis section to make this explicit. The QoR-15 sample size calculation is now presented only to demonstrate that the available sample was sufficient to detect the published MCID for this prespecified secondary analysis. (page 9, line 200)*

It’s hard to know what to make of this. If the authors are intentionally deceiving the reader into believing this is a new study, then this is academic fraud. However, the authors state “PACSPI-3 was conducted as a predefined sub-study embedded within the randomised, open-label clinical trial PACSPI-2.” Thus, I don’t think that the authors are trying to pretend that this is a new study. It would be helpful if the authors more clearly stated that this is an analysis of a previously published study.

*We appreciate the reviewer’s careful reading of our manuscript and are grateful that this concern was raised. We have revised the manuscript throughout to remove any ambiguity regarding the relationship between PACSPI-2 and the published study. We hope the revised version now clearly communicates that this manuscript reports a prespecified secondary analysis of the previously published PACSPI-2 randomised clinical trial.*

My other concern is simply that this isn’t a very interesting question. I’ve probably given anesthesiology to over 40,000 patients during my career. I’ve never provided anesthesia for a venous port insertion. This is a minimally invasive procedure typically performed with local anesthesia and light sedation performed by a surgeon and a nurse. It isn’t painful or traumatic. I have absolutely no idea why the authors would not have included this secondary endpoint in reference 13, which is their original study.

*We appreciate the reviewer's perspective. However, we believe that the present prespecified substudy addresses a distinct patient-centred research question that complements, rather than duplicates, the primary PACSPI-2 publication. The parent trial demonstrated that subcutaneous venous port implantation can be performed under local anaesthesia without increasing patients' intraprocedural pain compared with patient-controlled sedation. The present analysis extends those findings by examining whether the anaesthetic strategy influences patients' early postoperative recovery, assessed using the validated QoR-15 questionnaire. This outcome reflects aspects of recovery beyond procedural pain, including physical comfort, emotional state, psychological support, and functional recovery, and therefore provides additional clinically relevant information that was beyond the scope of the primary trial report.*

________________________________________

6. PLOS authors have the option to publish the peer review history of their article (what does this mean?). If published, this will include your full peer review and any attached files.

If you choose “no”, your identity will remain anonymous but your review may still be made public.

Do you want your identity to be public for this peer review? For information about this choice, including consent withdrawal, please see our Privacy Policy.

Reviewer #1: No

Reviewer #2: No

Reviewer #3: No

Reviewer #4: No

________________________________________

[NOTE: If reviewer comments were submitted as an attachment file, they will be attached to this email and accessible via the submission site. Please log into your account, locate the manuscript record, and check for the action link "View Attachments". If this link does not appear, there are no attachment files.]

To ensure your figures meet our technical requirements, please review our figure guidelines: https://journals.plos.org/plosone/s/figures

You may also use PLOS’s free figure tool, NAAS, to help you prepare publication quality figures: https://journals.plos.org/plosone/s/figures#loc-tools-for-figure-preparation.

NAAS will assess whether your figures meet our technical requirements by comparing each figure against our figure specifications.

*We assessed our figures accordingly.*

________________________________________

In compliance with data protection regulations, you may request that we remove your personal registration details at any time. (Remove my information/details). Please contact the publication office if you have any questions.
